# Important Limitations on the Conclusions of “Discrepant Results on Two Different Single Gene Noninvasive Prenatal Tests for Cystic Fibrosis: A Case Report”

**DOI:** 10.1055/a-2913-7060

**Published:** 2026-08-20

**Authors:** Julia Wynn, Jennifer Hoskovec

**Affiliations:** 1 692377 BillionToOne IncMenlo ParkCaliforniaUnited States

## 

We are writing to discuss the recent case report by Hogg describing a single discrepant single-gene noninvasive prenatal testing (sgNIPT) result in a pregnant patient with a fetus at high risk for cystic fibrosis (CF). 1 Several important considerations limit the conclusions that can reasonably be drawn from this report, and we believe these merit discussion to ensure the findings are interpreted in an appropriate clinical and scientific context.

Case reports serve a valuable purpose in identifying unusual or instructive clinical scenarios, but they are not an appropriate basis for drawing conclusions about the performance of an assay. The Unity assay (BillionToOne, Inc.) has been commercially available since 2019 and, over that time, has been used to screen hundreds of thousands of pregnancies. Its clinical performance for autosomal-recessive conditions has been characterized in multiple, independent, peer-reviewed publications in thousands of patients. Wynn et al. reported a sensitivity of 96.0%, specificity of 95.2%, and a negative predictive value (NPV) of 99.8%. 2 Hoskovec et al. similarly demonstrated high concordance between predicted fetal risk and neonatal outcomes with a sensitivity of 93.3% and NPV of 99.4%. 3 Wynn et al. further demonstrated that routine cfDNA prenatal screening with Unity successfully identified high-risk CF pregnancies in a real-world clinical setting, including cases that benefited from early prenatal intervention. 4 Importantly, these publications are in the test’s intended-use population, and they are in addition to Unity’s earlier clinical validity publications that had complete molecularly confirmed ground-truth data. 5 6

A single discordant result in one patient out of hundreds of thousands who have been tested neither contradicts nor meaningfully modifies these population-level estimates. False-negative results, which are expected in any screening test, are already explicitly captured in the published sensitivity data.

## The Validity of Newborn Screening as an Outcome Measure Deserves Clarification

The case report characterizes the Unity validation studies as limited on the basis that outcomes were collected using newborn screening (NBS) and the sample was enriched for patients with pre-test ( *a priori* ) general population risk and post-test sgNIPT high-risk results, which the author argues weakens the evidence. Neither of these points constitutes a rigorous critique of the study design. For rare autosomal-recessive conditions, the most analytically relevant outcomes are those that bear on sensitivity and positive predictive value: pregnancies with high-risk sgNIPT results and pregnancies with affected neonates. Requiring universal follow-up of all tested pregnancies to characterize performance of a rare-condition screen would introduce inefficiency, both time and cost, with minimal meaningful improvement in the precision of sensitivity or PPV estimates.

NBS is a highly sensitive assay designed to maximize detection and minimize missed cases for all conditions screened, and the methodology reflects this goal. Infants who screen positive for CF through NBS undergo confirmatory *CFTR* gene analysis. In practice, all cases of CF identified through NBS will have received a molecular genetic diagnosis by the conclusion of the NBS process. Therefore, the use of NBS outcomes does not represent a departure from molecular confirmation.

## The Two Assays are Held to an Unequal Evidentiary Standard

The author emphasizes the need for prospective studies with molecular genetic testing outcomes to validate sgNIPT. However, the clinical decision-making described in the report is difficult to reconcile with these evidentiary standards, as the author chose to use a novel platform, the Fetal Focus assay (Natera, Inc.), that had been released shortly before the clinical encounter without any peer-reviewed publication. Relying on a newly released test with no published peer-reviewed validation while simultaneously questioning the clinical validity of an established platform based on the very criteria of data availability is clearly inconsistent.

It is important to note that the Fetal Focus assay (Natera, Inc.), as of this writing, still has no published peer-reviewed clinical validation data. In contrast to the Unity studies, which were conducted in the intended-use population, the single ongoing study of the Fetal Focus assay is not. It is biased toward *a priori* high-risk couples, with the most recent limited read-out of the study, which is yet to be peer-reviewed, indicating an approximately 50× enrichment of affected cases compared with the general population. While it can be important to conduct prospective studies, it is more important for these studies to be in the intended-use population, especially when a methodology, such as that used in the Fetal Focus assay, is expected to benefit significantly from the availability of the partner sample. The author fails to mention this important limitation while criticizing the published studies of Unity.

## The Author Misrepresents the Record by Citing and Describing the False-Negative Literature without Its Context

The report cites three additional false-negative results from a letter by Zemanick et al. and draws on these cases to suggest a broader pattern of underperformance for the Unity assay. 7 However, the author fails to report Wynn et al.’s published response to Zemanick et al., which pointed out the statistical errors in Zemanick et al.’s calculations. 8 In a subsequent published response, Zemanick et al. explicitly clarified that their original letter “was never intended to address specific laboratory limitations.” Critically, Zemanick et al. agreed that “the initial risk assessments likely were incorrect due to the use of the wrong a priori risk” and stated directly that the three false-negative results “could have happened just by chance,” and do not reflect an error in the published metrics of the assay correcting their previous statement about the performance of the assay and highlighting that their primary goal was to ensure that all patients receive thorough counseling. 9 Citing the Zemanick letter without this subsequent discussion and clarification misrepresents the record.

## Conclusion

We share the author’s view that sgNIPT holds meaningful promise for prenatal care, particularly in cases where partner carrier screening is unavailable or incomplete, and we agree that patients and providers should receive thorough counseling on the benefits and limitations of all screening options. As with all prenatal screening technologies, these assays are designed to refine risk rather than to provide a diagnosis. The confirmatory amniocentesis in this case appropriately served as the basis for clinical decision-making and represents the recommended pathway for a diagnosis.

The Unity assay has been generating real-world performance data since 2019, with findings reported across multiple large peer-reviewed cohorts, while Fetal Focus entered clinical use only recently and without any comparable published evidence. A single true-positive result from a newly launched, unvalidated platform in one high-risk couple cannot be taken as evidence of superior performance relative to an assay with years of data and published validation.

As this field continues to evolve, well-designed prospective studies in the intended use population with robust outcome ascertainment will be essential for helping clinicians and patients make informed choices. We look forward to contributing to that evidence base and welcome rigorous scientific dialogue on how best to advance prenatal screening.
